# Anthropometric measures and nutritional status in a healthy elderly population

**DOI:** 10.1186/1471-2458-7-2

**Published:** 2007-01-03

**Authors:** Sergio Sánchez-García, Carmen García-Peña, María Ximena Duque-López, Teresa Juárez-Cedillo, Alma Rosa Cortés-Núñez, Sandra Reyes-Beaman

**Affiliations:** 1 Epidemiologic and Health Service Research Unit, Aging Area. XXI Century National Medical Center; Mexican Institute of Social Security, Mexico City, Mexico; 2 Nutritional Epidemiologic Research Unit. XXI Century National Medical Center; Mexican Institute of Social Security, Mexico City, Mexico; 3 Epidemiologic and Health Service Research Unit. Mexican Institute of Social Security, Querétaro, México; 4 Bedford Primary Care Trust, NHS, UK; 5 Unidad de Investigación Epidemiológica y en Servicios de Salud, Área de Envejecimiento. Edificio Administrativo. Tercer piso. Centro Médico Nacional Siglo XXI. Avenida Cuauhtémoc no. 330, Col. Doctores. Delegación Cuauhtémoc. México DF. Código Postal 06725. México

## Abstract

**Background:**

Anthropometric evaluation is an essential feature of geriatric nutritional evaluation for determining malnutrition, being overweight, obesity, muscular mass loss, fat mass gain and adipose tissue redistribution. Anthropometric indicators are used to evaluate the prognosis of chronic and acute diseases, and to guide medical intervention in the elderly. We evaluated anthropometric measurements and nutritional status as they relate to age and gender in healthy elderly people.

**Methods:**

The study analyzed data from the national survey "Health needs and health service use by older-than-60-year-old beneficiaries of the Mexican Institute of Social Security (IMSS)". The present study included only individuals who reported no chronic disease in the last 20 years and had no hospital admission in the two months prior to the survey. Anthropometric measurements included weight, height, body mass index (BMI), body circumference (arm, waist, hip and calf), waist to hip ratio (WHR), elbow amplitude and knee-heel length.

**Results:**

Application of the inclusion criteria resulted in a study population elderly of 1,968, representing 12.2% of the original number in the national survey in urban areas beneficiaries of the IMSS. The study population comprised 870 women and 1,098 men, with a mean age of 68.6 years. The average weights were 62.7 kg for women and 70.3 kg for men (p < 0.05), and the mean heights were 1.52 m for women and 1.63 m for men (p < 0.05). Age related changes in anthropometric values were identified. BMI values indicated that 62.3% of the population was overweight, and 73.6% of women and 16.5% of men had high fat tissue distribution.

**Conclusion:**

Our findings suggest that applying the BMI thresholds that identify being overweight in the general adult population may lead to an overestimation in the number of overweight elderly Similar problems appear to exist when assessing waist circumference and WHR values. Prospective studies are required to determine the associations between health and BMI, waist circumference and WHR in the elderly.

## Background

Anthropometric values are closely related to nutrition, genetic makeup, environmental characteristics, social and cultural conditions, lifestyle, functional status and health. Anthropometric evaluation is an essential feature of geriatric nutritional evaluation for determining malnutrition, being overweight, obesity, muscular mass loss, fat mass gain and adipose tissue redistribution. Anthropometric indicators are used to evaluate the prognosis of chronic and acute diseases, and to guide medical intervention in the elderly [1-3].

Anthropometric evaluation performed by trained health workers is inexpensive, non-invasive and provides detailed information on the different components of body structure, especially muscular and fat components, and can assist in assessing the nutritional status of a population [4]. Anthropometric measures are highly reliable for determining the nutritional status when compared with more sophisticated methodologies (hydrodensitometry, dilution techniques, measuring K-40 by whole body counting and electronic bioimpedance), the use of which is restricted by complexity and cost in population studies [5].

The aging process involves physiological and nutritional changes that are manifested by height and weight loss [6], muscular mass loss and fat mass increase. It also involves adipose tissue redistribution, with fat accumulation in the trunk and viscera.

Changes in body composition differ in men and women at different life stages and are reflected in anthropometric measures. Consequently, different anthropometric indicators are used at different life stages to evaluate the nutritional status. Some international studies in the older than 60 years population have investigated body composition changes [7-11] However there are no national Mexican references. Information on differences in body composition according to age and gender is also limited. Such information would be useful for correct nutritional evaluation of the elderly.

The present study evaluated anthropometric measures and nutritional status as they relate to age and gender in healthy elderly people with no chronic disease diagnosed in the last 20 years and no hospital admission in the two months prior to assessment.

## Methods

The "Health needs and health service use by older than 60 beneficiaries of the Mexican Social Security Institute (IMSS)" national survey was conducted from October 1996 to July 1997 [12]. The IMSS assists more than 40% of the Mexican population and more than 64% of the elderly population in Mexico, population covered by IMSS live mainly in urban areas. Methods and results are reported previously [13-15].

Briefly, subjects were selected randomly from those insured (not only users) at IMSS using a multi-stage sampling procedure. At the first stage two family medicine units were selected randomly in each Mexican state. Two consulting rooms were then selected randomly in each family medicine unit. Finally, households located in the geographical area covered by those consulting rooms were visited door by door to find people aged 60 years or over covered by IMSS.

The database generated by that survey was analyzed. Database includes information from the national level, nevertheless population from México City is underrepresented. The present study population comprised the 60-years-and-older IMSS beneficiaries with no chronic disease diagnosed in the past 20 years, and no hospital admissions during the two months prior to answering the survey. This elderly sample was considered because their favorable health condition allowed measurement of anthropometric parameters in the absence of influence from disease.

Exclusion criteria included cancer, diabetes mellitus, dyslipidemias, gout, arterial hypertension, heart attack, vascular brain events, chronic pulmonary obstructive disease, mental disorders, liver cirrhosis, gallbladder lithiasis, gastric or duodenal ulcer, chronic renal failure, kidney lithiasis, prostate hyperplasia, hip or femur fracture and other fractures.

The survey and anthropometric measurements were taken at subjects' homes in urban areas, by trained staff. The questionnaire included sociodemographic variables such as age, formal education, civil status, family situation and income. The measures analyzed were weight, height, body mass index (BMI), body circumferences (arm, waist, hip and calf), waist to hip ratio (WHR), elbow amplitude and knee-heel length [16,17].

### Anthropometric measurements

#### Weight

A portable scale with a 125 kg maximum capacity and a +/- 100 g error margin was used. Individuals removed shoes and heavy cloths prior to weighing.

#### Height

Subjects stood with their scapula, buttocks and heels resting against a wall, the neck was held in a natural non-stretched position, the heels were touching each other, the toe tips formed a 45° angle and the head was held straight with the inferior orbital border in the same horizontal plane as the external auditive conduct (Frankfort's plane).

#### Body circumferences

Mid-brachial, calf, waist and hip circumferences were measured using a flexible non-elastic measuring tape. Individuals stood with feet together and arms resting by their sides. The hip circumference was measured from the maximum perimeter of the buttocks. The waist circumference was taken as the plane between the umbilical scar and the inferior rib border. The waist circumference was used to identify individuals with possible health risks based upon threshold values of ≥ 88 cm for women and ≥ 102 cm for men [18].

#### Knee-heel length

This was determined using Chumlea's technique [19].

#### Body-mass index (BMI)

BMI was estimated by dividing weight (kg) by height^2 ^(m^2^) [20]. Individuals were considered malnourished if their BMI was less than 18.5, normal from 18.5 to 24.9 and overweight if ≥ 25 [21].

#### Waist to hip ratio (WHR)

This was estimated by dividing waist circumference by hip circumference [22]. The threshold WHR was ≥ 0.85 for women and ≥ 1.00 for men [23], above which superior distribution of adipose tissue was considered.

### Statistical methods

Data were recorded, validated and stored using the Statistical Package for the Social Sciences (SPSS) Windows software, version 11.0 [24]. The frequency and distribution of the characteristics of the elderly were analyzed using ANOVA to identify any age differences between anthropometric measurements in women and men and within the whole sample. Student's t test was use to evaluate differences between men and women according to age.

### Ethical approval

The original research proposal "Health Needs and Health Service Use by older than 60 beneficiaries of the IMSS" was approved by the Research and Ethic Committee, National Research Council at IMSS in 1996. Data were obtained under informed consent.

## Results

Of the 16,084 individuals who took part in the national "Health needs and health service use by older than 60 year old IMSS' beneficiaries" survey, 1,968 (12.2%) reported no chronic disease diagnosed in the past 20 years, and had no hospital admission up to two months prior to the survey. The present study analyzed this cohort, which comprised 870 women (44.2%) and 1,098 men (55.8%). There were not significant differences among the distribution by mexican state compared with the original sample.

The overall mean age was 68.6 (± 7.0) years; 67.8 (± 7.0) for women and 69.4 (± 6.8) for men. The age group with the largest number of individuals was the 60–64 years group (35.0% of the total population), followed by the 65–69 years group (26.8%).

The Education section of the survey showed that 27.2% of participants had never gone to school, 62.5% had at least one year of basic education and 10.4% had studied beyond elementary school. In terms of civil status, 73.0% were married or lived in free union, 21.6% were widows or widowers and 5.3% were single. In terms of family status, 54.5% belonged to a nuclear family, 32.4% lived in extensive family units and 0.8% lived alone. Earnings questions showed 83.1% had no income (Table 1).

**Table 1:** Characteristics of ≥ 60 year-old participating subjects.

	Gender					
	Women (n = 870)		Men (n = 1098)		Total (n = 1968)	
	
**Age**	N	%	n	%	n	%
60–64	371	42.6	317	28.9	688	35.0
65–69	209	24.0	319	29.1	528	26.8
70–74	141	16.2	217	19.8	358	18.2
75–79	91	10.5	152	13.8	243	12.3
80 and more	58	6.7	93	8.5	151	7.7
**Schooling**						
No schooling	265	30.5	271	24.7	536	27.2
Primary	527	60.6	703	64.0	1230	62.5
Secondary	36	4.1	72	6.6	108	5.5
Technical or professional	25	2.9	16	1.5	41	2.1
Preparatory	6	0.7	11	1.0	17	0.9
Professional	11	1.3	24	2.2	35	1.8
Postgraduate	0	0	1	0.1	1	0.1
**Civil status**						
Married	493	56.7	898	81.8	1391	70.7
Single	30	3.4	17	1.5	47	2.4
Separated	29	3.3	14	1.3	43	2.2
Free union	9	1.0	35	3.2	44	2.2
Divorced	8	0.9	10	0.9	18	0.9
Widow/er	301	34.6	124	11.3	425	21.6
**Family**						
Without information	69	7.9	53	4.8	122	6.2
Lives alone	8	0.9	7	0.6	15	0.8
Nuclear	439	50.5	633	57.7	1072	54.4
Multiple	27	3.1	38	3.5	65	3.3
Non family house	1	0.1	3	0.3	4	0.2
Extended	305	35.1	332	30.2	637	32.4
Combined	21	2.4	32	2.9	53	2.7
**Income**						
Yes	49	5.6	284	25.9	333	16.9
No	821	94.4	814	74.1	1635	83.1

Anthropometric values according to age and gender are shown in Table 2. Anthropometric measurements showed the age groups statistics differed in terms of weight, height, BMI, mid-brachial circumference, waist to hip ratio, waist circumference, hip circumference and elbow amplitude. Among women, age groups statistics differed in terms of weight, height, BMI, mid-brachial circumference, calf circumference, hip circumference and elbow width. In men, age groups statistics differed in terms of weight, height, BMI, mid-brachial circumference and elbow amplitude (p < 0.05).

**Table 2:** Anthropometric values according to age and gender participating subjects.

	Women	Men	Total		Women	Men	Total
	Mean ± S.D.	Mean ± S.D.	Mean ± S.D.		Mean ± S.D.	Mean ± S.D.	Mean ± S.D.
**Weight (kg)**^abc^				**Waist to hip ratio (WHR)**^c^			
60–64^d^	64.3 ± 11.7	71.8 ± 12.5	67.8 ± 12.6	60–64^d^	0.89 ± 0.08	0.95 ± 0.06	0.92 ± 0.08
65–69^d^	63.6 ± 10.4	71.8 ± 11.9	68.6 ± 12.0	65–69^d^	0.90 ± 0.07	0.94 ± 0.05	0.93 ± 0.06
70–74^d^	61.9 ± 11.1	69.6 ± 12.2	66.6 ± 12.3	70–74^d^	0.91 ± 0.10	0.95 ± 0.06	0.93 ± 0.08
75–79^d^	58.2 ± 11.7	67.7 ± 12.9	64.1 ± 13.2	75–79^d^	0.89 ± 0.07	0.95 ± 0.06	0.93 ± 0.07
80 and more^d^	57.4 ± 12.2	66.1 ± 10.4	62.8 ± 11.9	80 and more^d^	0.91 ± 0.10	0.96 ± 0.07	0.94 ± 0.09
Total^d^	62.7 ± 11.6	70.3 ± 12.3	66.9 ± 12.6	Total^d^	0.90 ± 0.08	0.95 ± 0.06	0.93 ± 0.07
**Height (cm)**^abc^				**Waist circumference (cm)**^c^			
60–64^d^	153.9 ± 7.2	163.8 ± 7.5	158.5 ± 8.8	60–64	93.8 ± 13.8	95.5 ± 11.6	94.5 ± 12.9
65–69^d^	152.6 ± 7.6	163.8 ± 8.1	159.4 ± 9.6	65–69	95.3 ± 14.0	96.1 ± 10.8	95.8 ± 12. 2
70–74^d^	151.3 ± 7.1	163.3 ± 8.7	158.6 ± 10.0	70–74	93.2 ± 11.9	95.4 ± 10.8	94.5 ± 11.3
75–79^d^	150.5 ± 7.4	161.3 ± 10.1	157.3 ± 10.5	75–79	91.1 ± 11.5	94.1 ± 12.5	93.0 ± 12.2
80 and more^d^	150.4 ± 8.2	162.0 ± 9.4	157.5 ± 10.6	80 and more	92.8 ± 15.1	95.7 ± 12.2	94.6 ± 13.4
Total^d^	152.6 ± 7.5	163.2 ± 8.5	158.5 ± 9.6	Total^d^	93.7 ± 13.4	95.5 ± 11.4	94.7 ± 12.4
**BMI (kg/m^2^)**^abc^				**Hip circumference (cm)**^a^^c^			
60–64	27.1 ± 4.5	26.8 ± 4.4	26.9 ± 4.4	60–64^d^	104.8 ± 12.8	100.4 ± 10.3	102.8 ± 11.9
65–69^d^	27.3 ± 4.2	26.7 ± 3.9	26.9 ± 4.1	65–69^d^	105.3 ± 13.0	101.1 ± 9.6	102.8 ± 11.2
70–74	27.0 ± 4.4	26.0 ± 4.1	26.4 ± 4.3	70–74^d^	102.5 ± 11.5	100.1 ± 9.6	101.1 ± 10.5
75–79	25.5 ± 4.2	26.0 ± 5.0	25.8 ± 4.8	75–79	101.6 ± 10.9	98.8 ± 10.4	99.9 ± 10.6
80 and more	25.2 ± 4.4	25.3 ± 5.0	25.3 ± 4.7	80 and more	101.6 ± 14.5	98.8 ± 10.2	99.8 ± 12.1
Total^d^	26.8 ± 4.4	26.4 ± 4.4	26.6 ± 4.4	Total^d^	104.0 ± 12.6	100.2 ± 10.0	101.9 ± 11.4
**Mid-braquial circumference (cm)**^abc^				**Elbow amplitude (cm)**^abc^			
60–64	30.5 ± 4.0	30.8 ± 4.0	30.7 ± 4.0	60–64	27.8 ± 12.3	29.4 ± 11.5	28.6 ± 11.9
65–69	29.8 ± 4.5	30.4 ± 4.1	30.1 ± 4.3	65–69	28.6 ± 11.8	28.6 ± 12.5	28.6 ± 12.2
70–74	30.1 ± 4.2	29.5 ± 3.8	29.7 ± 4.0	70–74	24.6 ± 10.3	26.0 ± 13.7	25.4 ± 12.5
75–79	29.2 ± 4.7	28.6 ± 3.7	28.8 ± 4.1	75–79	25.3 ± 13.7	25.8 ± 14.1	25.6 ± 13. 9
80 and more	27.3 ± 5.7	28.8 ± 3.9	28.2 ± 4.7	80 and more	23.9 ± 11.9	24.5 ± 12.0	24.3 ± 11.9
Total	29.9 ± 4.4	29.9 ± 4.0	29.9 ± 4.2	Total^d^	27.0 ± 12.1	27.6 ± 12.7	27.3 ± 12.5
**Calf circumference (cm)**^a^				**Knee-heel length (cm)**			
60–64	35.2 ± 8.1	35.8 ± 8.0	35.5 ± 8.1	60–64^d^	46.2 ± 4.4	50.0 ± 5.6	47.9 ± 5.3
65–69	34.8 ± 9.2	35.6 ± 8.6	35.3 ± 8.8	65–69^d^	46.3 ± 3.5	49.5 ± 5.9	48.2 ± 5.4
70–74	33.7 ± 7.4	34.9 ± 9.1	34.4 ± 8.5	70–74^d^	46.4 ± 4.1	50.0 ± 4.1	48.6 ± 4.5
75–79	34.1 ± 9.2	33.6 ± 8.7	33.8 ± 8.9	75–79^d^	46.6 ± 4.0	50.2 ± 5.0	48.8 ± 4.9
80 and more^d^	31.3 ± 4.2	34.7 ± 8.7	33.4 ± 7.5	80 and more^d^	46.3 ± 4.9	50.0 ± 3.9	48.6 ± 4.6
Total	34.5 ± 8.3	35.2 ± 8.6	34.9 ± 8.4	Total^d^	46.3 ± 4.1	49.9 ± 5.2	48.3 ± 5.1

^a ^Significant differences between age range in women (p < 0.05)

^b ^Significant differences between age range in men (p < 0.05)

^c ^Significant differences between age range (p < 0.05)

^d ^Significant differences between women and men (p < 0.05)

Gender comparisons showed weight, height, waist to hip ratio, waist circumference, elbow amplitude and knee-heel length were greater in men, while BMI and hip circumference were greater in women (p < 0.05). Men and women did not differ in terms of mid-brachial and calf circumferences.

For each age group, men had greater weight, height, waist to hip ratio and knee-heel length than women. In the 70–74 years age group, the mean BMI was larger for women than men. In the 80-years-and-older age group, men had a larger calf circumference than women. In the 60–64, 65–69 and 70–74 years age groups, women had a greater hip circumference compared to men (p < 0.05). No statistics differences were found between genders in terms of mean mid-brachial circumference, hip circumference and elbow width in any age group.

BMI was used to determine malnutrition and overweight (Table 3). Malnutrition was found in 1.4% of the population (< 18.5 BMI); with 1.6% of women and 1.2% men being malnourished. Malnutrition was observed in 0.8% of 60–64 year-olds and 3.3% of > 80 year-olds. We found that 62.3% of the population was overweight (BMI ≥ 25.0; 65.4% of women and 59.9% of men).

**Table 3:** Body-mass index (BMI) according to age and gender in ≥ 60 year-old participating subjects.

		Edad											
				
		60–64 years		65–69 years		70–74 years		75–79 years		80 year and more		Total	
Sex	BMI	n	%	n	%	n	%	n	%	n	%	n	%
Women	<18.5	4	1.1	2	1.0	4	2.8	2	2.2	2	3.4	14	1.6
	18.5 – 24.99	123	33.2	57	27.3	42	29.8	39	42.9	26	44.8	287	33.0
	≥25	244	65.8	150	71.8	95	67.4	50	54.9	30	51.7	569	65.4
													
Men	<18.5	3	0.9	2	0.6	3	1.4	2	1.3	3	3.2	13	1.2
	18.5 – 24.99	110	34.7	117	36.7	91	41.9	66	43.4	43	46.2	427	38.9
	≥25	204	64.4	200	62.7	123	56.7	84	55.3	47	50.5	658	59.9
													
Total	<18.5	7	1.0	4	0.8	7	2.0	4	1.6	5	3.3	27	1.4
	18.5 – 24.99	233	33.9	174	33.0	133	37.2	105	43.2	69	45.7	714	36.3
	≥25	448	65.1	350	66.3	218	60.9	134	55.1	77	51.0	1227	62.3

Waist circumference was used to identify individuals with possible health risks. We found that 68.9% of women had a waist circumference ≥ 88 cm, and 26.1% of men had a waist circumference ≥ 102 cm (Table 4). In addition, 73.7% of women and 19.1% of men (WHR ≥ 0.85 and WHR ≥ 1.0) showed central adipose tissue distribution. For every age group, men had a lower frequency of central adipose tissue distribution than women (Table 4).

**Table 4:** Waist circumference and waist to hip ratio (WHR) according to age and gender in ≥ 60 year-old participating subjects.

**Waist circumference (cm)**												
	60–64 years		65–69 years		70–74 years		75–79 years		80 years and more		Total	
	N	%	N	%	n	%	N	%	n	%	n	%

Women												
≥88	262	70.6	146	69.9	100	70.9	55	60.4	36	62.1	599	68.9
<88	109	29.4	63	30.1	41	29.1	36	39.6	22	37.9	271	31.1
												
Men												
≥102	84	26.5	85	26.6	55	25.3	32	21.1	31	33.3	287	26.1
<102	233	73.5	234	73.4	162	74.7	120	78.9	62	66.7	811	73.9

**Waist to hip ratio (WHR).**												

Women												
≥0.85	258	69.5	160	76.6	107	75.9	69	75.8	47	81.0	641	73.7
<0.85	113	30.5	49	23.4	34	24.1	22	24.2	11	19.0	229	26.3
												
Men												
≥1.00	65	20.5	51	16.0	43	19.8	26	17.1	25	26.9	210	19.1
<1.00	252	79.5	268	84.0	174	80.2	126	82.9	68	73.1	888	80.9

Of overweight women (BMI ≥ 25), 74.7% had a WHR ≥ 0.85, while by comparison, only 23.1% (n ≥ 152) of overweight men (BMI ≥ 25) had a WHR ≥ 1.00 (Table 5).

**Table 5:** Distribution of body-mass index (BMI) and waist to hip ratio (WHR) participating subjects.

WHR	BMI					
	
	<18.5		18.5 – 24.99		≥25	
Women	n		n		n	
≥0.85	5	35.7	80	27.9	144	25.3
<0.85	9	64.3	207	72.1	425	74.7
Total	14	100.0	287	100.0	569	100.0
Men						
≥1.00	1	7.7	57	13.3	152	23.1
<1.00	12	92.3	370	86.7	506	76.9
Total	13	100.0	427	100.0	658	100.0

## Discussion

In general, body mass increases during adulthood and decreases progressively with old age at a rate of approximately one kilogram per decade. Furthermore, during old age, height is estimated to decrease at 0.5 – 1.5 cm per decade [25]. Mean weight and height are both greater in men than women, and both gradually decrease as age advances in both men and women [26]. Consistent with those findings, the present study found that height decreased with age. As age advances, the skeletal system undergoes structural modifications such as demineralization, which reduces the width of vertebrae and deforms the long bones of the inferior extremities.

Velázquez-Alva and colleagues [27] studied 508 retired and pensioned older-than-60-years individuals ascribed to the IMSS and the National Institute for Elderly Population (INSEN) living in Mexico City. They found that in women, the average age, weight and height was 67.3 ± 6.8 years, 60.8 ± 9.9 kg and 149.9 ± 5.9 cm, respectively, while for men it was 66.9 ± 6.42 years, 70.7 ± 9.92 kg and 163.8 ± 5.53 cm, respectively. These values are similar to those found in the present study, particularly for men.

In both clinical practice and epidemiology, BMI is the most used indicator to determine both the individual and collective general nutritional status. This index is considered to positively correlate with certain health and longevity indicators [28,29]. In the present study, malnutrition as determined using the BMI (< 18.5) was observed in 1.4% of the population, and was higher in women (1.6%) than in men (1.2%). While it has been reported that a high percentage of the elderly are malnourished in developed countries [30], the present results do not reflect this possibly because the present study population did not include individuals diagnosed with chronic-degenerative or acute diseases. Studies in elderly hospitalized populations show a higher percentage of malnutrition and weight loss a month prior to hospital admission. Thus, weight loss and malnutrition may increase the risk of hospital admission [31].

Some authors have indicated that BMI thresholds should be modified for the elderly population. Sergi et al recommended as threshold to malnutrition in the elderly a BMI < 20.0 [32]. Using this cut-off point 4.5% of our population have malnutrition. However, we consider that in order to validate such a threshold in Mexican population, further investigation is necessary. Risk factors, mortality trends, and nutritional and biochemical markers are indeed important to research.

Other suggested that a BMI between 25.0 and 29.9 should be considered desirable (5). Such a modification would result in many elderly currently classified as overweight being re-categorized as normal. It is necessary consider the convenience of adopt this cut-off point, since a BMI under this point has been set as a protective factor for mortality and morbidity in chronic-degenerative diseases [2,26,29,33-36]. However, health results suggest the desirable BMI range is greater in the elderly compared to young adults. This difference relates to body composition changes in old age. With this cut-off point in this population BMI ≥ 30.0 [10], that percent of overweigh could be 19.4%, also a more realist proportion (21.5% women and 17.7% men).

We cannot ignore the difficulties associated with obtaining a precise and reliable height measure in the elderly. This problem could be solved by using alternative measurements such as knee height, or another index such as weight/knee height, which may be more appropriate. The prognostic value of this index needs to be evaluated to determine appropriate interpretation methods [37].

A study in Chicago involving 3,981 men and 3099 women investigated the impact of nutritional status on the quality of life in people > 65 years old. Obesity (BMI ≥ 30) was associated with lower physical and social performance in women only, but had no mental impact. Being overweight (BMI 25.0 – 29.9) was associated with a decrease in physical wellbeing in women only. Low weight (BMI < 18.5) in men and women was associated with decreased physical, social and mental wellbeing. Both overweight and low weight values were associated with a lower quality of life, worse physical performance and less physical wellbeing. Those results question the acceptance of a BMI from 25.0 to 29.9 as being normal for the elderly [38].

Another study highlighted the importance of weight in the elderly in regard to function and mobility, regardless of weight and nutritional status upon arriving at old age [37]. That study recommended overweight elderly should be advised to maintain weight, or undergo weight loss strategies accompanied by physical activity to help preserve fat-free mass. Strategies aiding adults to attain old age with a healthy BMI may not only reduce the risk of later functional and mobility deficits, but may also prevent morbidity and mortality associated with cardiovascular diseases.

A study carried out in Mexico City by Velázquez-Alva et al. [27] reported men and women had a similar mean brachial circumference across age groups, consistent with the present findings. In contrast, the mean calf circumference in the present study was greater than that reported in the study by Velázquez-Alva and colleagues.

In the general adult population, a waist circumference ≥ 88 cm in women and ≥ 102 cm in men is a health risk indicator in relation to BMI and WHR, with these values associated with a sensitivity of greater than 94% and a specificity of 97% [18]. Thus, this may be a good option for determining the BMI and WHR parameters in a fast and reliable manner. A study carried out in Holland found that in the general adult population, individuals with a waist circumference higher than the above-mentioned thresholds have an overload of factors that placed their health at risk [39]. The present study of the elderly found that 68.9% of women and 26.1% of men had waist circumferences above the threshold values, suggesting elderly women may be at greater risk than elderly men in terms of health. This trend was observed across age groups. Further studies are needed to examine whether waist circumferences of ≥ 88 cm for women and ≥ 102 cm for men are indeed health risk indicators in the elderly.

The ratio between waist circumference and hip circumference (WHR) is being used more frequently to estimate possible relative increases in abdominal fat in order to identify individuals at risk of developing non-insulin-dependant diabetes mellitus, dyslipidemias, arterial hypertension and coronary artery disease [34-36,40-42]. In the general adult population, WHR values of < 1.00 for men and < 0.85 for women are considered desirable [43], and individuals with values above these are at greater risk for cardiovascular disease morbidity and mortality. However, the present results indicate that, at least in elderly women, these thresholds should be re-evaluated. Using BMI and WHR in combination could assist health professionals in assessing the nutritional status of the elderly, and assist in implementing the necessary measures to control obesity in the elderly with high adipose tissue distribution.

Anthropometric methods for assessing the nutritional status in adults are simple, inexpensive and potentially reliable. However, problems arise when evaluating elderly populations as there is limited information to interpret anthropometric data in this age group. Reference values for the evaluation of the nutritional status in older adults have been based upon extrapolations from studies using young adults or based on statistical definitions of threshold values, rather than on population studies on elderly morbidity, mortality and quality of life. It is necessary to consider all of these factors to determine desirable threshold values for anthropometric measures in the elderly population.

The lack of anthropometric cross-sectional surveys nationality in the elderly population in Mexico limits the comparison of our gender and age-specific results with those produced by other studies.

## Conclusion

Our findings suggest that applying the BMI thresholds that identify being overweight in the general adult population may lead to an overestimation in the number of overweight elderly. Also, it seems that could be an underestimation of malnutrition. Similar problems appear to exist when assessing waist circumference and WHR values. Prospective studies are required to determine the associations between health and BMI, waist circumference and WHR in the elderly. Further investigation is necessary to validate the BMI thresholds in Mexican population.

## List of abbreviations

IMSS: Mexican Institute of Social Security

BMI: body mass index

WHR: waist to hip ratio

INSEN: National Institute for Elderly Population

## Competing interests

The author(s) declare that they have no competing interests.

## Authors' contributions

SSG originated the idea for this study, did the research proposal, data analysis and prepared the manuscript. CGP contributed to the research proposal, reviewed the analysis and participated in the manuscript preparation. MXDL participated in the data analysis and in the interpretation of the anthropometric measures and she also participated in writing the paper. TJC participated in the interpretation of the data and in the discussion of the paper. ARCN participated in the research proposal and reviewed the manuscript. SRB designed and conducted the original proposal., and was involved in the data analysis and in the preparation and discussion of the manuscript.

All authors approved the final version.

## Pre-publication history

The pre-publication history for this paper can be accessed here:


